# Emerging Role of Enhancer RNAs as Potential Diagnostic and Prognostic Biomarkers in Cancer

**DOI:** 10.3390/ncrna8050066

**Published:** 2022-10-01

**Authors:** Somayeh Panahi-Moghadam, Shokoufeh Hassani, Shirin Farivar, Faezeh Vakhshiteh

**Affiliations:** 1 Department of Genetics, Faculty of Biological Sciences, Tarbiat Modares University, Tehran 1411713116, Iran; 2 Department of Cell and Molecular Biology, Faculty of Life Sciences and Biotechnology, Shahid Beheshti University, Tehran 1983969411, Iran; 3 Department of Toxicology and Pharmacology, Faculty of Pharmacy, Tehran University of Medical Sciences, Tehran 1417614411, Iran; 4 Toxicology and Diseases Group, Pharmaceutical Sciences Research Center (PSRC), The Institute of Pharmaceutical Sciences (TIPS), Tehran University of Medical Sciences (TUMS), Tehran 1417614411, Iran; 5 Oncopathology Research Center, Iran University of Medical Sciences (IUMS), Tehran 1449614535, Iran

**Keywords:** enhancer RNA (eRNA), cancer, prognosis, diagnosis

## Abstract

Enhancers are distal *cis*-acting elements that are commonly recognized to regulate gene expression via cooperation with promoters. Along with regulating gene expression, enhancers can be transcribed and generate a class of non-coding RNAs called enhancer RNAs (eRNAs). The current discovery of abundant tissue-specific transcription of enhancers in various diseases such as cancers raises questions about the potential role of eRNAs in disease diagnosis and therapy. This review aimed to demonstrate the current understanding of eRNAs in cancer research with a focus on the potential roles of eRNAs as prognostic and diagnostic biomarkers in cancers.

## 1. Introduction

Enhancers are distal *cis*-acting elements that are known to regulate gene expression via spatial chromatin loops formation with target promoters [1,2]. They are short (50–1500 bp) regulatory elements of accessible DNA that assist in regulating the cell transcriptional machinery through increasing the transcription of target genes. Structurally, enhancers are open/accessible chromatin with low levels of DNA methylation, which are bound by RNA polymerase II (RNApol II), transcription factors (TFs), and cofactors, particularly transcription initiation factors, such as TBP, TFII, and P300/CBP. Enhancers are flanked by histones with permissive chromatin markers of histone H3 lysine 27 acetylation (H3K27ac) and histone H3 lysine 4 methylation (H3K4me) [3,4,5,6,7]. While promoters are *cis*-acting elements that recruit transcription in a position- and direction-dependent manner, enhancers perform freely of their position and orientation regarding their target gene; consequently, these elements can establish physical communication to interact distant promoters. Rather than contributing to gene expression, enhancers can be dynamically transcribed, forming a class of non-coding RNAs known as enhancer RNAs (eRNAs). It was initially anticipated that the product of enhancer transcription is the noisy outcome of the transcription procedure. Nevertheless, later studies suggest various roles for eRNA as a universal cellular mechanism involved in directing cell characteristics and function. In this review, we demonstrate recent understanding of eRNA structure along with function. We further exhibit the latest studies regarding the role of eRNAs in cancer with emphasis on eRNA as prognostic and diagnostic biomarkers in cancer. We also provide a brief overview on data resources to explore eRNAs in cancer research. 

## 2. Biogenesis and Function of eRNA

**Figure 1:**
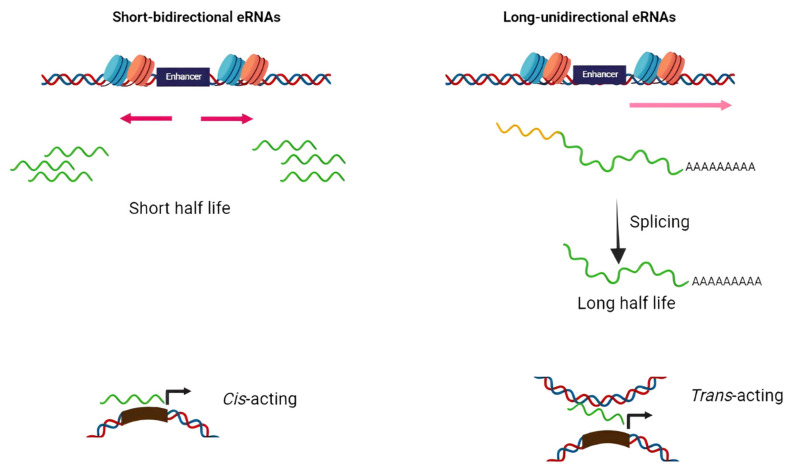
Schematic diagram of two distinct classes of eRNAs. The majority of eRNAs are short, bidirectional, non-polyadenylated, and unstable while others are long unidirectional, polyadenylated, and more stable. The former has *cis*-acting action while the latter perform as *trans*-acting elements.

Based on structure and transcription patterns, eRNAs (approximately from 0.1–9 Kb) [8] can be classified in two groups of short bidirectional, non-spliced, non-polyadenylated RNAs and long unidirectional, spliced, stable polyadenylated transcripts (Figure 1). Since eRNAs are mainly non-polyadenylated and unstable, they are predominantly localized in the nucleus and chromatin-enriched fractions [9,10,11,12]. Transcription of eRNAs generally occurs prior to mRNA expression from the target gene [13,14,15,16]. The tissue-specific transcription of enhancers has been shown in various diseases such as cancers. Enhancers typically contain specific DNA elements that are recognized by tissue-specific TFs. These factors often cooperate in their binding to enhancers and frequently synergize to achieve the optimal activation of target genes [17]. Under extracellular stimuli and the activation of specific signaling pathways, TFs are recruited into the enhancer region, bind to particular DNA sequences, and stimulate the remodeling of nucleosome and histone modifications (regions enriched by H4K8ac, H3K27ac, and H3K4me are hallmarks of active enhancers) [18,19,20]. H3K27 and H4K8 are acetylated through CBP histone acetyltransferases, and p300 and chromatin is further opened in the enhancer region and, thus, RNApol II and BRD4 cofactor are recruited to the enhancer [20]. Integrator, a large complex associated with the carboxyl-terminal domain (CTD) of RNApol II, has an important role in transcriptional termination at the enhancers. The depletion of the integrator leads to the reduction in processed eRNAs and accumulation of primary eRNA transcripts [21].

**Figure 2:**
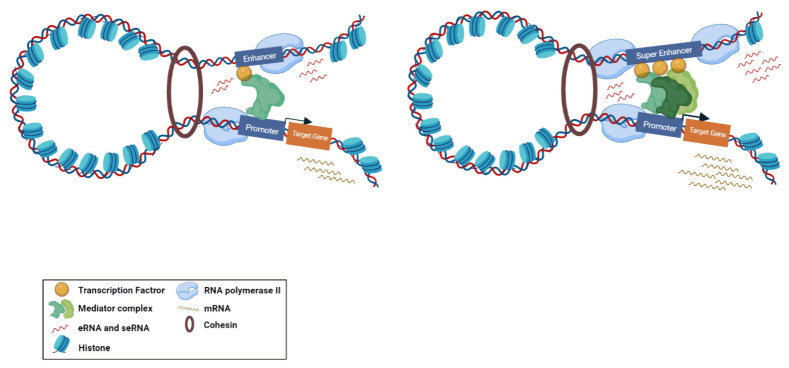
Biogenesis of typical eRNA and seRNA and their corresponding function. Active enhancers are bidirectionally transcribed to produce eRNAs and seRNAs. Super enhancers are augmented with higher amount of transcription factors, mediators, and RNApol II compared to enhancers. Therefore, the transcription activity at a super enhancer is typically higher than at a distinct enhancer. From the functional perspective, super enhancers have a greater potential to stimulate target gene transcription.

Super-enhancers (SE) are described as a cluster of enhancers that have dense assemblies of RNApol II, TFs, and typical enhancer histone modifications (H4K8ac, H3K27ac, and H3K4me) that leads to a greater amount of super-enhancer RNA (seRNA) production (Figure 2) [22]. The difference between conventional enhancers and SEs is clearly displayed in the nature of the dependence of the transcription activity ensured by the regulatory element and the number of TFs and cofactors associated with it [23]. The transcription activity at an SE is typically higher than at a distinct enhancer. SEs have high potential to activate the transcription of their target genes and play significant roles in tissue-specific biological processes [24]. Most SE produce unidirectional polyadenylated seRNAs, which are more stable and have a longer half-life than non-polyadenylated eRNAs [25]. Production of different isoforms by alternative splicing and *cis* and *trans* actions of seRNAs can orchestrate a precise pattern of gene expression [25,26]. As previously mentioned, eRNAs were initially considered as transcriptional noise of enhancers. Later, by using experimental methods, including global run-on sequencing (GRO-seq), Start-seq, and CRISPR/Cas9, several investigations have revealed that a subclass of eRNAs contribute to enhancer function, especially the regulation of gene expression [27,28,29]. As most eRNAs are unstable, their recognition is mainly proceeded via precision nuclear run-on sequencing (PRO-seq), GRO-seq, chromatin immunoprecipitation (ChIP-seq) [30,31,32], or cap analysis of gene expression (CAGE) sequencing [33] rather than the common RNA-seq. Using the CRISPR-Display method, eRNAs were demonstrated to bind to catalytically dead Cas9 (dCas9) for targeting a particular locus of a genome [34]. In another approach, single-molecule fluorescence in situ hybridization (smFISH) [35,36] and ChIRP-seq [37,38] were used as powerful methods for detection of eRNA loci in the genome. Overexpression and knockdown studies of eRNAs demonstrated that this group of non-coding RNAs have strong correlation with their target mRNAs [39]. This correlation is largely dependent on the proximity and correct interactions between the enhancer and promoter. Moreover, chromatin interaction studies revealed that enhancer–promoter looping structure induces higher expression of eRNAs in comparison with other enhancer regions [40,41]. Some studies suggested that eRNAs can act as a *cis* regulatory element and initiate or stabilize enhancer–promoter looping through association between TFs, mediators, cohesins, and RNApol II [42,43]. Moreover, eRNAs were shown to function in *trans* for modifying the chromatin structure and directing chromatin accessibility at protein-coding promoter regions [44]. The interaction of eRNAs with CBP and p300 histone acetyltransferases were shown to have a prominent impact on the modulation of H3K27 acetylation and methylation as eRNA knockdown led to decreased levels of H3K27ac and increased levels of H3K27me3 at target-promoter regions [45,46,47,48]. Upon interaction with enhancers, Polycomb repressive complex 1 and 2 (PRC1 and PRC2) have been shown to play regulatory roles in Polycomb-mediated gene transcription [49,50]. Although PRC1 and PRC2 have gene repression activities, in some cases it has been proposed that Polycomb chromatin domains can affect gene expression by forming chromatin topologies that support gene induction [51]. PRC2, for instance, composed of the EZH2 and SUZ12 subunits, which is responsible for establishing and maintaining histone H3K27 methylation during cell differentiation. The interaction of eRNAs and the EZH2 subunit of the PRC2 complex represses its methyltransferase activity and consequently leads to reduced H3K27me3 level and increased gene expression [1,39]. Direct interaction of eRNA with RNApol II, TFs, and general cofactors was shown to be required for initiation and elongation of transcription [14,52]. NELF and P-TEFb complexes are negative and positive elongation factors, respectively, which are released and recruited to RNApol II in the elongation phase. eRNA interacts with NELF and P-TEFb and further promotes the release of paused RNApol II and transition to active elongation by acting as decoys for these complexes [14,47].

## 3. Functional Roles of eRNAs in Cancer

Given that enhancers are recognized to influence the maintenance of different types of cells, it is not unexpected that their malfunction has emerged as a powerful factor behind numerous types of malignancies. Translocation, duplication, insertion, deletion, or point mutation at enhancer regions, and especially transcription factor binding elements [53,54], are frequently observed in cancers [55,56]. One interesting possibility is that these types of mutations make a difference in eRNA expression that eventually drives cancer development. For instance, specific three-stranded nucleic acid organization of the DNA:RNA hybrid and the related non-template single-stranded DNA, known as R-loop, can be shaped at enhancer regions with exceeded eRNA expression levels. Particularly, R-loops are correlated with genomic instability and DNA injury, proposing an association in the initiation and progression of cancer [57]. Moreover, single-stranded DNA (ssDNA) in R-loops may be an off-target for the action of the activation-induced cytidine deaminase (AID) enzyme [58]. Intrinsically, this enzyme is responsible for initiating somatic hypermutation on ssDNA at immunoglobulin (Ig) loci and preferentially alters cytosine to uridine by deamination [59]. AID off-target positions correlate with extremely transcribed enhancers, which promotes genome instability and tumorigenesis [60]. 

Several studies uncover roles for individual eRNAs in tumorigenesis of many cancer types, including ovarian, breast, prostate, colorectal, and lung adenocarcinomas, showing that their ectopic expression is strongly linked to enhancer dysfunction [61,62,63]. In tumor cells, eRNAs regulate target genes by both *cis*- and *trans*-regulatory activities and, hence, play a crucial role in a variety of important signaling cascades [37,64]. For instance, in colorectal cancer, it has been stated that the presence of Colon Cancer-associated Transcript 1 (*CCAT1*) eRNA was highly correlated with *c-Myc* overexpression [63]. MYC is accepted as a crucial regulator of cell proliferation and deregulation of this proto-oncogene associated with the development of many cancer types [65]. In a separate study, the knockdown of oncogenic *CCAT1* eRNA in squamous cell carcinomas suppressed the SE-associated genes expression required for the propagation and migration of cancer cells [66]. *Net1e* eRNA, which is located downstream of *NET1* proto-oncogene, is a breast cancer specific eRNA and its knockdown by LNA (locked nucleic acids) antisense RNA was shown to strongly reduce cell proliferation in the MCF7 breast cancer cell line [67]. *ARIEL* in leukemia [68], *HPSE* in different cancer types [69], and *P2RY2* in bladder cancer [70] are other examples of eRNAs targeted by knockdown approaches that may serve as new therapeutic targets for cancer treatment. In breast cancer, 17b-oestradiol (E2)-bound estrogen receptor α (ER- α) could raise the expression of enhancers close to E2-induced coding genes. These differentially expressed eRNAs were demonstrated to elevate the strength of ER-α activated looping of the enhancer–promoter by direct interaction with cohesin. Targeted knockdown of eRNA from corresponding enhancers attenuated cohesion attachment to the ER-α enhancer and consequently reduced enhancer–promoter looping [37]. Wang et al. indicated that *WAKMAR2* can be a new candidate eRNA in modulating the microenvironment of invasive breast cancer cells and its downregulation might influence the immune-related genes expression in favor of tumor progression. eRNAs are implicated in various cancer signaling pathways by potentially modifying their target genes, such as immune checkpoints and clinically actionable genes [71]. By successful delineation of basic eRNA mechanisms, including RNA–RNA, RNA–DNA, and RNA–protein interactions, these eRNAs can be considered as new therapeutic targets and will pave the way for eRNA-based cancer diagnostic and therapeutic approaches [72].

## 4. Data Resources to Explore eRNA in Cancer

As mentioned before, most eRNAs are unstable and non-polyadenylated with low abundance [2]. Thus, they are not easily detectable in routine RNA-sequencing methods, which are based on polyadenylated RNAs. Alternative techniques rely on measuring promising transcripts, such as global run-on sequencing (GRO-Seq) [7], precision run-on nuclear sequencing (PRO-Seq) [73], and cap analysis gene expression (CAGE) [33] to certify that no eRNA is missed. These methodologies are instrumental for the detection of formerly undiscovered eRNAs and active enhancers. For example, the CAGE technique was applied by the FANTOM consortium for profiling the large amounts of transcriptomes of different types of cells, from which 43,011 enhancer elements were revealed to be transcribed to eRNAs [74]. Since the number of detected eRNA transcripts are increased exponentially, comprehensive databases and computational pipelines are highly required to illustrate and consolidate the eRNA expression profiles in normal and cancerous samples. Currently, two types of eRNA data resources were generated. While datasets such as Ensemble (https://www.ensembl.org, accessed on 1 January 2002), ENCODE (https://www.encodeproject.org, accessed on 5 September 2012), FANTOM (http://fantom.gsc.riken.jp/index.html, accessed on 26 March 2014), and the Roadmap Epigenomics Project (http://www.roadmapepigenomics.org, accessed on 13 October 2010) include numerous annotated regulatory elements containing enhancers, other datasets such as The Cancer Genome Atlas (TCGA) (https://portal.gdc.cancer.gov, accessed on 26 September 2013) and Genotype-Tissue Expression (GTEx) (https://gtexportal.org/home, accessed on 29 May 2013) have multi-omic data including RNA-seq and survival data from patient samples and the Cancer Cell Line Encyclopedia (CCLE) (https://portals.broadinstitute.org/ccle/about, accessed on 8 May 2019) that apply genomics and sequencing data in ~1000 cancer cell lines for pan-cancer and tumor-specific analysis of eRNAs. These omics data can be downloaded via Xena platform. UCSC Xena cancer browser (https://xena.ucsc.edu, accessed on 22 May 2020) allows biologists to correlate between genomic and/or phenotypic variables with visualizations and analyses. To facilitate research on eRNA, many enhancer pipelines such as SEdb, HACER, RAEdb, HEDD, DiseaseEnhancer, TiED, SEA, and DENdb [75,76,77,78,79,80,81] have been generated. GeneHancer [82] is one of the most common pipelines, which integrates the enhancer annotations from four altered enhancer resources, including Ensembl, FANTOM, VISTA, and ENCODE [10,83,84,85]. Human enhancer RNA Atlas (HeRA) is another data portal that accommodates data from the ENCODE, FANTOM, and GTEx that presents an expression profile and regulatory network of eRNAs in normal human samples [86]. On the contrary, the eRic (eRNA in cancer) database (https://hanlab.uth.edu/eRic, accessed on 8 October 2019) can predict eRNA functions in cancer via collecting eRNA expression profiles, clinical features, target genes, and drug response [67]. By using RNA-seq data from TCGA and GTEx and using CAGE-defined enhancers annotated by FANTOM, Chen et al. developed The Cancer eRNA Atlas (TCeA) data portal, which provides a high-resolution map of eRNA loci. In this map, SE showed discrete loci with sharp eRNA expression peaks. The annotation of SE activities can be used for a broad range of biomedical investigations, such as immunotherapy response and enhanced explanations of cancer phenotypes by resolving intratumoral heterogeneity [87].

## 5. eRNAs as Prognostic and Diagnostic Biomarker in Cancer

Even though remarkable progress has been made in the field of cancer research, there are still a number of issues that need to be improved, such as delayed diagnosis and poor prognosis. Non-coding RNAs have gained wide consideration in recent years because of their specific expression and functional diversity in a variety of cancers [88]. They play critical roles in various biological pathways and hold great promise in cancer diagnosis and therapy. Clinical trials have also initiated investigating non-coding RNA-based medications as adjuncts to traditional chemotherapeutics [89]. Regarding eRNAs, an increasing number of studies have reported that these non-coding RNAs have amenable prognostic and diagnostic values due to their tumor-specific expression patterns [90,91]. In this section, we will review the present findings on eRNAs and their potential prognostic and diagnostic values in cancers. Table 1 summarizes eRNAs and seRNAs as diagnostic and/or prognostic biomarkers in different cancers.

### 5.1. Head and Neck Squamous Cell Carcinoma

Head and neck cancer is considered one of the most common malignancies in the world, with ~870,000 new cases and ~440,000 deaths in 2020 [92] in which the most common histological subtype of head and neck cancer is head and neck squamous cell carcinoma (HNSCC). Feng et al. showed the role of certified eRNAs as an innovative biomarker in HNSCC. The group indicated the role of eRNA in 500 HNSCC cases by means of an eRNA expression matrix annotated from the TCGA database. Functional enrichment analyses were carried out using Gene Ontology and the Kyoto Encyclopedia of Genes and Genomes (KEGG). Global expression of eRNAs was increased in tumor tissues compared to normal cases; out 369 differentially expressed eRNAs, 330 were upregulated and 39 were downregulated. According to the eRNA expression matrix and survival information, 5 eRNAs were identified with a correlation with the prognosis value in HNSCC cases, which represent an innovative finding in the molecular mechanisms of HNSCC [93]. Gu et al. demonstrated the role of prognosis-related *AP001056.1* eRNA in HNSCC. In this study, an incorporated data analysis methodology was developed to recognize major eRNAs in HNSCC. To discover the RNA levels and clinical data from the TCGA project, the interactive web servers, TANRIC (the Atlas of Noncoding RNAs in Cancer) and cBioPortal were applied. From the obtained 5 significant eRNA candidates, *AP001056.1* was the most significant survival-associated eRNA in HNSCC with immune-related *ICOSLG* as its target gene. While strong associations between *AP001056.1* and *ICOSLG* expression were demonstrated in a number of cancers, the most significant effect on overall survival (OS) was observed in HNSCC [94]. 

**Table 1:** eRNAs and seRNAs as diagnostic and/or prognostic biomarkers.

Cancer Type	eRNAs/seRNAs	Deregulation in Cancer	Target Gene/Pathways	Clinical Sample/Number of TCGA Cases	Sample/Model Information	Application	Ref.
HNSCC	*ENSR00000188847* *ENSR00000250663* *ENSR00000313345* *ENSR00000317887* *ENSR00000336429*	Up	-	500 TCGA HNSCC samples	Patient sample	Prognosis	[93]
	*AP001056.1*	Down	*ICOSLG*	426 TCGA HNSCC samples	Patient sample	Prognosis	[94]
LUAD	*TBX5-AS1*	Down	*TBX5*	10 LUAD samples	Patient sample	Prognosis/Diagnosis	[95]
	188 functional eRNAs	129 Up/59 Down	Cell cycle and immune system-related pathways	80 LUAD samples/481 TCGA LUAD samples	Patient sample	Prognosis	[62]
CRC	*CCAT1* *CCAT2*	Up	*c-Myc*	150 CRC samples	Patient sample	Prognosis	[96]
	*RP11-569A11.1*	Down	*IFIT2*	39 CRC samples	Patient sample/cell line	Diagnosis	[97]
	*PVT1*	Down (epigenetic regulation mediated through aberrant methylation in CRC)	*Myc*	698 TCGA CRC dataset	Patient sample	Prognosis	[98]
GC	*EMX2OS*	Up	*EMX2*	375 TCGA GC samples	Patient sample	Prognosis	[99]
	*FALEC*	Up	*ECM1*	60 GC samples	Patient sample/cell line	Prognosis	[100]
	*HPSE*	Up	hnRNPU/p300/EGR1/HPSE axis	90 GC samples	Patient sample/cell line	Prognosis	[69]
	*CDK6-AS1*	UP (in patients below 60 years)	*CDK6*	407 TCGA GC samples	Patient sample	Prognosis	[101]
	*WAKMAR2*	Down	*TNFAIP3*	371 TCGA GC samples	Patient sample	Prognosis	[102]
Breast Cancer	*SLIT2*	Down	MAPK/c-Fos signaling pathway	1211 TCGA breast cancer and 12 bone metastases samples	Patient sample/cell line	Prognosis/Bone metastasis	[103]
	*WAKMAR2*	Down	*IL27RA* *RAC2* *FABP7* *IGLV1-51* *IGHA1* *IGHD*	1104 TCGA invasive breast cancer samples	Patient sample	Prognosis	[71]
HCC	*DCP1A*	Up	*PRKCD*	1580 TCGA samples together with 1791 target genes	Patient sample	Prognosis	[104]
	*SPRY4-AS1*	Up	*SPRY4*	124 TCGA samples	Patient sample	Prognosis	[105]
	*AL445524.1*	Up	*CD4-CLTA4* related genes	371 TCGA HCC tumor samples and 54 adjacent normal specimens	Patient sample	Prognosis	[106]
Brain Cancer	*AC003092.1*	Up	*TFPI2*	161 TCGA GBM patients	Patient sample	Prognosis	[107]
	*CYP1B1-AS1*	Up	*CYP1B1*	10,000 TCGA cancer sufferers covering 33 diverse cancer types	Patient sample	Prognosis	[108]
	*LINC00844* *MRPS31P5* *CRNDE*	Down Down Up	*PHYHIPL* *ATP7B* and *NEK3* *IRX5*	693 TCGA cohorts and 325 cohort in Chinese Glioma Genome Atlas (CGGA)/40 glioma samples	Patient sample	Prognosis/Diagnosis	[109]
	*ENSR00000210436* *ENSR00000249159* *ENSR00000195717* *ENSR00000195824* *ENSR00000094845* *ENSR00000283518* *ENSR00000094854* *ENSR00000031043* *ENSR00000031044* *ENSR00000260651* *ENSR00000146066* *ENSR00000301859* *ENSR00000213692* *ENSR00000326719* *ENSR00000134110* *ENSR00000134111* *ENSR00000134112* *ENSR00000013533* *ENSR00000013524* *ENSR00000082228* *ENSR00000048324* *ENSR00000082228* *ENSR00000048324*	Association with immune-related dysfunctions in the TME	*ADCYAP1R1* *FGF13* *PSMB8* *MAPT* *BMPR1A* *DDX17* *ELN* *BMP2* *SEMA6C* *PDIA2* *PTPN6* *SSTR5* *CD4*	TCGA and CGGA samples	Patient sample/cell line	prognosis	[110]
Prostate Cancer	*K-KLK3*	Up	*KLK3*	45 patient samples	Patient sample/cell line	Diagnosis	[111]
	PARGP1	Up	AGAP4	TCGA database	Patient sample	Prognosis	[112]
Bladder Cancer	*MARC1*	Up	−	37 tissues	Patient sample/cell line	Diagnosis	[113]
	*EMP1*	UP	APOLD1 and GPRC5A/ KRAS signaling, etc.	411 TCGA bladder urothelial carcinoma samples	Patient sample	Prognosis/Bone metastasis prediction	[114]
ESCA	*AC007255.1*	Up	*PRR15*	162 ESCA TANRIC database/12 pairs of ESCA tissues and normal tissues	Patient sample	Prognosis	[115]
Colon Adenocarcinoma	*LINC02257*	Up	*DUSP10*	521 TCGA samples	Patient sample	Prognosis	[116]
Ovarian Cancer	*FOXP4-AS1*	Down	*FOXP4*	379 TCGA samples/42 patient samples	Patient sample	Prognosis	[117]
Thyroid Cancer	*NBDY* *MEG3* *AP002358.1* *AC141930.1*	Relation to the prognosis of thyroid cancer patients	*-*	510 TCGA samples	Patient sample	Prognosis/Diagnosis	[90]
PAAD	*LINC00242*	Down	*PHF10*	177 PAAD data set from UCSC	Patient sample/cell line	Prognosis	[91]

Abbreviations: HNSCC, head and neck squamous cell carcinoma; LUAD, lung adenocarcinoma; CRC, colorectal cancer; GC, gastric cancer; HCC, hepatocellular carcinoma; ESCA, esophageal cancer; PAAD, pancreatic adenocarcinoma.

### 5.2. Lung Cancers

In a comprehensive analysis conducted by Cheng et al., *TBX5-AS1* eRNA was identified as a potential prognostic/diagnostic biomarker and therapeutic target for lung adenocarcinoma (LUAD) patients. According to the results, *ATBX5-AS1* and *TBX5,* as its regulatory target gene, were meaningfully downregulated in tumor cases in comparison with matched-adjacent pairs. The pan-cancer validation outcomes indicated that *TBX5-AS1* was linked to survival in four tumors, namely, adrenocortical carcinoma (ACC), LUAD, lung squamous cell carcinoma (LUSC), and uterine corpus endometrial carcinoma (UCEC). *TBX5-AS1* and *TBX5* showed a correlation in 26 tumor types. The group anticipated that in the early stage of the disease, the expression level of eRNA *TBX5-AS1* in blood or other body fluids, can be detected to demonstrate if the patient has LUAD. Furthermore, the prognosis of cases that have been identified with LUAD might be predicted according to TNM stage and the levels of *TBX5-AS1* expression in blood [95]. 

The inclusion of “gender” as a research variable still has several challenges, nonetheless, researchers should always be aware of bias toward gender differences and discover possible mechanisms to address this. Given the significance of gender differences in oncology, Yan et al. intended to monitor eRNAs in patients with non-small cell lung cancer (NSCLC). The results showed that *TBX5-AS1* eRNA was differently expressed between female and male cases. The strong indication evidenced that male patients with a high expression of *TBX5-AS1* showed a malignant immune microenvironment with urgent need for immune checkpoint inhibitor treatment. Therefore, the prognostic significance of *TBX5-AS1* eRNA was observed only in male cases with lung squamous cell carcinoma (SCC) type and provide a reference for immunotherapy [118]. Qin et al. demonstrated functional molecular characteristics of eRNAs and the clinical value of eRNA-based molecular subtypes in LUAD. A total of 3297 eRNAs were identified from whole-genome sequencing and RNA-seq data of 80 LUAD patients, which were altogether upregulated in tumor specimens compared to non-cancerous tissues. The prognostic value of the eRNA-based molecular subtypes was additionally assessed among 481 TCGA LUAD specimens. Accordingly, 188 functional eRNAs were identified, and consensus clustering of these eRNAs proposed an innovative biological aspect complementary to other genomic characteristics. The results of this study further provided clinical implications for LUAD treatment [62]. 

### 5.3. Colorectal Cancer

Thean et al. investigated if *CCAT1* and *CCAT2* expression was coordinately upregulated in colorectal cancer (CRC) and whether their upregulation was associated to that of their target *c-Myc* in CRC samples. *CCAT1* and *CCAT2* are two enhancer-derived long non-coding RNAs (lncRNAs) located approximately 500 and 300 kb, respectively, upstream of their oncotarget, *c-Myc*. Upregulation of these eRNAs were connected to worse prognosis in CRC. Expression of *CCAT1*, *CCAT2*, and *c-Myc* were considerably upregulated in the tumors in comparison with matched pairs. Further, *c-Myc* and *CCAT2* levels in the tumor were likewise significantly increased in metastatic compared to non-metastatic cases. The levels of *CCAT1* and *CCAT2* in both the matched mucosa and the tumors were not meaningfully associated to time to metastasis; however, the levels of *c-Myc* in the tumors as well as the matched mucosa was significantly associated to time to metastasis. Therefore, *CCAT1* and *CCAT2* involvement in metastasis incidence was negligible and might be accounted for through their impacts on the regulation of their oncotarget *c-Myc* [96]. The latest investigations have shown that super-enhancer-associated lncRNAs (SE-LncRNAs) act as critical players in carcinogenesis. Chen et al. identified a new SE-LncRNA, *RP11-569A11.1*, and investigated its functional role in the progression of CRC. From a total of 23 differentially expressed SE-LncRNAs in CRC tissues, *RP11-569A11.1* was demonstrated to be significantly decreased in CRC cells and specimens. Further analyses indicated a positive regulatory association between *RP11-569A11.1* and its target gene, *IFIT2*. Therefore, *RP11-569A11.1* was shown to inhibit CRC tumorigenesis via the IFIT2-dependent pathway and may be considered as a potential diagnostic biomarker in CRC [97]. Shigeyasu et al. demonstrated the role of *PVT1* in CRC. Using the genome-wide FANTOM enhancer database, the *PVT1* locus exhibited oncogenic enhancer activity and was shown to target the oncogene *Myc*. High levels of the *PVT1* transcription from the PVT1 locus was connected to poor survival in CRC patients in stage II and III. The findings suggested that the *PVT1* level is a favorable prognostic biomarker and a promising therapeutic target in CRC [98].

### 5.4. Gastric Cancer

Liu et al. investigated the role of prognosis-related eRNAs and related target genes in gastric cancer (GC) patients. Using Kaplan–Meier correlation analysis and co-expression analysis from 63 eRNAs associated with prognostics in GC, the topmost 6 eRNAs of *LINC01714*, *ZNF192P1*, *AC079760.2*, *LINC01645*, *EMX2OS*, and *AC114489.2* were identified. The top *EMX2OS* eRNA was demonstrated to be associated with OS in GC patients and has the potential to be an innovative therapeutic target in clinical practice. The correlation analysis confirmed that eRNA *EMX2OS* is associated with age, tumor stage, size, and histological grade. The pan-cancer analysis revealed that *EMX2OS* was correlated with poor survival consequences in several cancer types [99]. Wu et al. demonstrated the role of *FALEC* eRNA in GC. *FALEC* with proximity to the tumor metastasis-associated extracellular matrix protein 1 (*ECM1*) gene was indicated to be significantly elevated in GC samples compared to paired adjacent non-cancerous specimens. In addition, expression of *ECM1* and *FALEC* were connected positively, and elevated expression of *ECM1* anticipated shorter survival time in patients. Based on the outcomes, *FALEC* downregulation was considerably shown to inhibit the invasive potential of GC cells via interfering *ECM1* expression. The results of this study suggested a promising biomarker and therapeutic target for personalized management of GC patients [100]. Jiao et al. demonstrated the promoting role of *HPSE* eRNA in progression of cancer. In clinical specimens from 90 primary GC patients, higher expression of *HPSE* eRNA was noted when compared to the normal gastric mucosa, which was positively linked to *HPSE* expressions. Likewise, increased expression of *HPSE* eRNA was associated with *HPSE* levels in prostate cancer, cervical cancer, and hepatocellular carcinoma patients with shorter OS time. Hence, *HPSE* eRNA could be considered as a prognostic factor for poor outcome in cancer cases [69]. Yang et al. identified *CDK6-AS1* eRNA as a prognostic biomarker in GC with positive association with *CDK6* as its target gene. *CDK6-AS1* might be regarded as a prospective biomarker for GC and a predictor for sensitivity to chemotherapeutic drugs [101]. Zhang et al. investigated the relation of *WAKMAR2* eRNA expression and prognosis of GC. Based on the findings, patients with high levels of *WAKMAR2* demonstrated a promising prognosis compared to cases with low *WAKMAR2* levels. Thus, *WAKMAR2* is considered as a favorable biomarker that can be regarded as an OS predictor in GC patients [102].

### 5.5. Breast Cancer

eRNAs were shown to be engaged in tumor progression and metastasis. Li et al. demonstrated the role of eRNAs in breast cancer bone metastasis. According to the co-expression network for bone metastasis of breast cancer-associated eRNAs, crucial eRNAs were screened to explore a prognostic model to predict the bone metastasis. In this regard, 27 hub eRNAs were nominated, and a survival-associated linear risk assessment model with a relatively high accuracy was developed. Furthermore, immune-related eRNAs of *SLIT2*, *CLEC3B*, *LBPL1*, *FRY*, *RASGEF1B*, *DST*, and *ITIH5* as prognostic signatures for bone metastasis of breast cancer were confirmed by Least Absolute Shrinkage and Selection Operator (LASSO), multivariate Cox regression, and Cell-type Identification by Estimating Relative Subsets of RNA Transcripts (CIBERSORT) analysis. The outcomes of this study provided bioinformatics data in offering the molecular mechanisms of the metastasis to the bone. Furthermore, the possible regulatory signaling pathway of *SLIT2* in breast cancer bone metastasis was identified providing a favorable therapeutic strategy for metastasis of breast cancer [103]. Wang et al. investigated the eRNAs that play a role in immune responses in invasive breast cancer. Accordingly, eRNAs that were associated with the survival of breast cancer patients were screened and selection was performed based on immune-related genes as targets for constructing an eRNA-immune gene regulatory network. According to the finding, *WAKMAR2* was acknowledged as a main candidate engaged in invasive breast cancer through modulating the tumor microenvironment and will possibly function by regulating immune-related target genes, including *IL27RA*, *RAC2*, *FABP7*, *IGLV1-51*, *IGHA1*, and *IGHD*. *WAKMAR2* was demonstrated to be involved in tumor immunity and revealed decreased levels in invasive breast cancer specimens. This study approved *WAKMAR2* as a promising molecular marker for the prognosis of individuals with invasive breast cancer and provided indication for novel probable targets for breast cancer therapy [71].

### 5.6. Hepatocellular Carcinoma

Wu et al. investigated the expression level and prognostic significance of fundamental eRNAs in hepatocellular carcinoma (HCC). Related data of 1580 eRNAs together with 1791 target genes were obtained from the TCGA-LIHC gene expression database. Among the identified eRNAs, *DCP1A* was the utmost prognosis-related eRNA that presented the maximum association with *PRKCD* as its target gene. According to the results, the poor OS in HCC specimens was associated with excessive expression levels of *DCP1A* eRNA and *PRKCD*. The upregulation of *DCP1A* was correlated with advanced size, grade, and stage status. The results of pan-cancer analysis further indicated that the expression of *DCP1A* was differentially expressed in 13 other tumor types, especially in digestive system tumors [104]. In a similar study conducted by Ye et al., 124 prognosis-related eRNAs involved in HCC progression were identified by analyzing TCGA datasets. The findings suggested a SPRY4 antisense RNA 1 (*SPRY4-AS1*) eRNA as a potential biomarker and therapeutic target for HCC. Elevated expression of *SPRY4-AS1* was linked to poor prognosis of HCC patients as the results confirmed that the *SPRY4-AS1* eRNA was overexpressed in HCC samples. The pan-cancer data revealed that *SPRY4-AS1* was linked to several types of cancer. Moreover, positive relations were detected between *SPRY4-AS1* and its target gene, *SPRY4*, in 16 types of malignancies [105]. Cai et al. explored the participation of eRNAs in the development of malignancies by targeting immune checkpoints and tumor-related genes. The group intended to create an immune-related eRNA prognostic model that may be used to prospectively evaluate the prognosis of patients with HCC. A total of 64 immune-related eRNAs were recognized in HCC, of which 14 were linked to OS. Of these eRNAs, 5 were applied to construct an immune-related eRNA signature, showing an association with poor survival and to be an independent prognostic biomarker for HCC. Expressions of these 5 eRNAs in tumor specimens were compared to adjacent non-cancerous specimens. According to the results, the *AL445524.1* eRNA was demonstrated to perfume as an oncogene that influences prognosis somewhat by modulating CD4-CLTA4 related genes. Consequently, *AC007255.1* might be considered as a prognostic marker for HCC patients [106].

### 5.7. Brain Tumors

Guo et al. recognized prognosis-related eRNAs and related estimated target genes in glioblastoma multiforme (GBM). RNA-seq data from GBM cases were obtained via TCGA. Among them, 70 eRNAs were acknowledged as prognosis-related genes, demonstrating substantial correlation with OS. The results of this study suggested that *AC003092.1*, an immune-related eRNA, was associated with a glioma-immunosuppressive microenvironment, which indicated important association with survival in GBM specimens and the upregulated expression of target gene, *TFPI2*. The association was confirmed in the levels of *AC003092.1* and *TFPI2* in high-grade gliomas as well as GBM cell lines [107]. Ye et al. intended to detect the survival-related eRNAs and to investigate their probable role in GBM. Thus, RNA-sequencing data were obtained from TCGA datasets for 31 types of tumors and 74 survival-related eRNAs were identified with a focus on *CYP1B1-AS1*, which showed the maximum cor value. KEGG analyses recommended that *CYP1B1-AS1* be considered as a critical eRNA that can be involved in cancer development via CK-CKR mutual effect, TNF and JAK-STAT signaling pathways, and complement and coagulation cascades. In 29 out of 31 tumor types, the positive correlation was documented between *CYP1B1-AS1* and its target gene, *CYP1B1*. The findings evidenced that overexpression of *CYP1B1-AS1* may be a promising biomarker to predict prognosis of GBM patients [108]. Lin et al. demonstrated the association of eRNAs and brain tumors. Using data retrieved from the TCGA and CGGA (Chinese Glioma Genome Atlas) databases, the hub eRNAs correlated to glioma prognosis were determined. Gene expression analysis was performed on the glioma samples to assess the expression features of the identified hub eRNAs. Selected were 3 prognosis-associated eRNAs, and a risk signature that can predict survival outcomes for glioma patients was constructed. Furthermore, pathways and biological functions linked to the risk signature were investigated based on hub eRNAs and their target genes. Notably, the risk signature showed the potential to be utilized as a biomarker for glioma diagnosis and therapy [109]. Tian et al. targeted identification of survival-mediating immune-related genes controlled by eRNAs and/or seRNAs and discovered possible mechanisms causing immune failure in the tumor microenvironment of gliomas. Among the 13 immune-related genes identified, 8 were regulated, particularly by eRNA or seRNA. Herein, the identified immune-related genes were used, and a prognostic tool was constructed to predict survival outcomes. The result demonstrated how exact eRNAs and seRNAs were essential elements affecting survival outcomes. The obtained results might be considered to progress the therapeutic outcomes and extend the OS of glioma patients as well as other cancer types [110]. 

### 5.8. Prostate Cancer

Nishimura et al. investigated the role of eRNAs as potential diagnostic markers in prostate tumors. In this study, the expression profile of the *KLK3* eRNAs, as possible markers of cancer progression, were examined in prostate cancer cells and samples. Accumulated evidence supported that prostate tumor expansion may correlate with epigenetic reorganization in the *KLK3* genomic regulatory elements reproduced by alterations of the *KLK3* eRNA expression. Thus, the *KLK3* eRNAs levels were indicated to be dissimilar in distinct tumor specimens and, furthermore, varied between normal and cancerous parts from the same tissue specimens [111].

In a similar study conducted by Ang et al., 12 eRNAs associated with prostate cancer survival was identified using TCGA. Among them, *PARGP1* was demonstrated as the most clinically significant eRNA with an association with recurrence in prostate cancer. Expression levels of *PARGP1* significantly differed between patients with prostate-specific antigen (PSA) values from 0–4 ng/mL and from 4–10 ng/mL, suggesting that *PARGP1* can be applied as a promising diagnostic marker to distinguish benign from malignant lesions when the PSA is less than 10 ng/mL [112]. Pan et al. investigated whether the sense and antisense eRNA was controlled by androgen receptor (AR) action in prostate cancer cells. While a decline in antisense eRNA reduced its neighbor mRNA levels, tumor progression, and invasion, the levels of antisense eRNA were associated with recurrence and clinical marker PSA levels in patient samples. The results showed that the functional antisense eRNA might be a potential target for prostate cancer diagnosis and therapy upon suppression. Novel chromatin collaboration among enhancer, promoter, and gene-ending regions may offer innovative vision into the spatiotemporal mechanism of the gene transcription and action of bi-directional eRNAs [119].

### 5.9. Bladder Cancer

Liu et al. examined the oncogenic role of androgen-associated androgen receptor (AR) mediated-eRNA *MARC1* in the development of bladder cancer. According to the findings, *MARC1* eRNA expression was higher in the bladder cancer specimens compared with adjacent non-tumor tissues. Furthermore, its upregulation was positively associated with poor survival in bladder cancer cases. Therefore, the potential role of *MARC1* as a biomarker for the diagnosis of bladder cancer was implicated [113]. Hao et al. identified eRNAs for the prognosis of bone metastasis in bladder cancer. The RNA-seq information of patients with bladder urothelial carcinoma was retrieved from TCGA databases. The differentially expressed eRNAs were recognized between primary bladder cancer with positive or negative bone metastasis. The prognostic bone metastasis-specific eRNAs were selected by Lasso regression and prediction models were constructed. Finally, the possible mechanism in bladder cancer bone metastasis together with prognostic biomarkers and therapeutic targets were explored. The results provided a prediction model that identified eRNAs as trustworthy indexes for prognosis and bone metastasis prediction in bladder urothelial carcinoma patients [114].

### 5.10. Esophageal Cancer 

Wang et al. demonstrated prognostic eRNAs and the related target genes in esophageal cancer (ESCA). Among 2695 lncRNAs that transcribed from active enhancer regions, 33 were considerably associated to OS. The group found that there was a positive correlation between *AC007255.1* levels and pathological TNM, stage, and grade with negative correlation with OS time. The findings demonstrated that *AC007255.1* and its potential target, *PRR15*, were both elevated in esophageal cancer specimens. Therefore, *AC007255.1* eRNA was meaningfully correlated to esophageal cancer patients’ prognosis [115].

### 5.11. Colon Adenocarcinoma

Xiao et al. revealed the role of eRNAs in colon adenocarcinoma. Using large-scale data mining, the role of 39 eRNA genes with association with colon adenocarcinoma prognosis were identified, amongst which 25 eRNAs indicated considerable relations with their expected target genes. *LINC02257* was recognized as the substantial survival-associated eRNA, with *DUSP10* as its target gene. Furthermore, the high expression of *LINC02257* in colon adenocarcinoma was more vulnerable to unfavorable prognosis and correlated with various clinical characteristics. The co-expression of *LINC02257* and its prospective signaling pathways over diverse cancer types suggested that *LINC02257* could perform as an independent predictive biomarker via the PI3K-Akt signaling pathway for colon adenocarcinoma [116]. 

### 5.12. Ovarian Cancer

Hua et al. demonstrated the significance of eRNAs in ovarian cancer. Using the UCSC Xena platform, the gene expression pattern and clinical data of ovarian cancer and several other types of cancers were obtained. In total, 71 eRNA candidates revealed significant association with OS of ovarian cancer specimens, of which 23 were identified to be associated with their potential target genes. The expression of forkhead box P4 antisense RNA 1 (*FOXP4-AS1*) had the maximum positive association with *FOXP4*, its expected target gene. The results of this study proposed that *FOXP4-AS1* eRNA levels might be a promising independent prognostic biomarker candidate in ovarian cancer [117].

### 5.13. Kidney Renal Clear Cell Carcinoma

Jiang et al. investigated the role of eRNAs in kidney renal clear cell carcinoma (KIRC). Gene expression profiles and clinical data of KIRC and 32 other types of cancer were acquired using the Xena platform. Enhancer RNAs and their target genes were selected as putative enhancer RNA-target pairs. *EMX2OS* was explored as the eRNA most correlated with OS in KIRC patients. The downregulation of *EMX2OS* was meaningfully correlated with histological grade, stage, and prognosis. Based on the findings of this study, *EMX2OS* eRNA showed positive effects on survival and can be regarded as a new beneficial target in KIRC [120].

### 5.14. Thyroid Cancer

Liang et al. identified a four-eRNA-based prognostic signature of *AC141930.1*, *NBDY*, *MEG3*, and *AP002358.1* in thyroid cancer for survival prediction. The gene expression profile and clinical data of thyroid cancer were obtained from the genotype-tissue expression (GTEx) and TCGA databases and was subjected to differential and cluster investigation. According to the results, the risk assessment model based on the four eRNAs can predict the prognosis of thyroid cancer patients with good accuracy [90]. 

### 5.15. Pancreatic Adenocarcinoma

Tong et al. demonstrated that eRNA *LINC00242* transcribed from the enhancer area of *PHF10* was meaningfully associated to patients’ survival in pancreatic adenocarcinoma (PAAD). According to the results, decreased expression levels of *LINC00242* were associated with poor clinicopathological characteristics. Knockdown evaluation of *LINC00242* by siRNA transfection suggested that *PHF10* is a downstream target gene of *LINC00242*. *PHF10* levels were discovered to considerably correlate with the immune cell infiltration and immune subtype in several kinds of cancer. The outcomes of this study offered an approach for predicting the prognosis of patients with pancreatic adenocarcinoma as well as a potential target for immunotherapy [91].

## 6. Conclusions and Future Direction

According to the investigational analysis of histone modifications, chromatin accessibility, cap structures, and chromatin conformations, it is anticipated that more than six million enhancers exist in the human genome. eRNAs are products of active enhancers and their expression is strongly tissue-specific and tightly regulated according to their critical role in the regulation of target gene transcription and the maintenance of the cell state. Current advances show that aberrant regulation of eRNAs is strictly linked to tumorigenesis. Cancer specificity, noninvasiveness, and relative ease in detection make eRNAs suitable cancer biomarkers. eRNA expression was demonstrated to be extensive but specific to tumor kinds and individual patients, therefore, can be considered as potential diagnostic/prognostic markers or therapeutic targets. Consequently, it was concluded that the field of enhancer-derived non-coding RNAs hold considerable therapeutic potential and warrants additional research with respect to cancer diagnosis, prognosis, and therapeutic strategies. Although remarkable progress has been made in this field, several issues need to be addressed. The majority of eRNAs are still poorly characterized with regard to function, interactions with protein-coding genes, and mechanisms that modulate their expression. The short half-life and chromatin association are the common features that make eRNAs and seRNAs difficult to characterize. Development of sensitive techniques to identify eRNA targets would also be essential. Multiple models have been proposed to explain the mechanisms underlying the functions of eRNA, including the in *trans* mechanism by recruiting co-activators to activate nearby genes, and the in *cis* mechanism via facilitating looping and trapping TFs to the enhancer locus to keep chromatin accessible and active. However, additional experiments are needed to completely cognize the underlying mechanisms of eRNAs in gene regulation. Consistent and comprehensive genome-wide annotations of eRNAs across various types of cells will be essential to dissect the mechanisms and to guide the field in discovering significant features of eRNAs. Furthermore, developing in vivo imaging approaches to screen the collaboration dynamics between eRNA and locus in genomes would facilitate recognizing the sequential events during transcriptional activation. Addressing these issues will shed new light on the full concern of the chief roles of enhancer elements in gene regulation, homeostasis, and diseases. In the future, an in-depth understanding of the mechanism by which eRNAs and seRNAs modulate gene expression and their contribution to disease pathogenesis may assist in recognizing new therapeutic targets and malignancies biomarkers.
